# A Case of Double Amputation of the Leg for Diabetic Gangrene

**Published:** 1906-06

**Authors:** Herman E. Hayd

**Affiliations:** Buffalo, N. Y.; Surgeon to the German and the German Deaconness’s Hospitals. 493 Delaware Avenue


### CLINICAL REPORTS.

### A Case of Double Amputation of the Leg for Diabetic Gangrene.

By HERMAN E. HAYD, M. D., Buffalo, N. Y.
Surgeon to the German and the German Deaconness's Hospitals.

Gangrene of the lower extremeties is not an uncommon complication in diabetes, but when it involves both feet, it is sufficiently rare and interesting to be worthy of a report in the JOURNAL, and particularly when the patient operated upon was sixty-eight years of age.

Operations on persons the subject of diabetes have always been looked upon as serious undertakings, but I am persuaded that these dangers have been much exaggerated when the patients are over fifty years of age, because the condition is then the result of more or less pronounced angiosclerosis, particularly in the terminal vessels.

Diabetes in a young person is a very serious matter, but when the subject is advanced in years, the disease can exist and often does exist without producing any marked or distressing symptoms, and its presence is often never suspected and not found out until the urine is examined for some other purpose, such as a life insurance application or in the course of some other disease.

The history of the following case proves that large operations can be undertaken under these circumstances, and that suffering can be relieved and life prolonged, when they are performed before the system is too much weakened and depleted, and the mere presence of sugar, even in large amounts, does not contraindicate any kind of surgical interference when it is strongly indicated.

Miss R——, 68 years of age, of Scotch origin, came under my care in June 1905, and was suffering from gangrene of the fourth toe of the right leg. The urine contained over 4% of sugar, acid in reaction, specific gravity 1046, four pints per diem, no acetone or diacetic acid. She was dieted and given large doses of codeia, but her pain was so excruciating and her condition was growing progressively worse, hence an operation was recommended, and on October 21, 1905, at the German Deaconness's Hospital, the leg was amputated below the knee joint. The flaps healed with only a little sloughing on the anterior skin flap, and a splendid stump resulted.

The condition of the vessel was interesting. The artery was tied off just before the division took place and its coats were markedly atheromatous. It was brittle and crackled under the fingers, but its lumen was of fair size. She left the hospital in about six weeks in excellent physical condition. Her diet was very nutritious and general; in fact, she was permitted to eat anything she desired, and she was given a bottle of Bass's ale every day.

Two months after the first operation, the same suspicious discoloration was seen on the fourth toe of the left leg, and she complained of great pain in the ball of the foot. The pain continued with great severity, and the discoloration advanced, so that it was quite evident we were dealing with a progressive gangrene. She was advised to have the leg amputated, which was done on January 29, 1906, and she left the Hospital in nine or ten weeks.

Union of the flaps did not take place by first intention; the wound opened and the tissues sloughed very badly. There was very little constitutional disturbance; the temperature never went above 102 degrees, nor the pulse 110. Gradually the slough was thrown off and the ends of the bones became covered with healthy granulations, but finally skin formed over them, so that the resulting stump is very good. The artery was very much thickened and stood out like a tooth pick and broke like a piece of clay pipe.

This case is interesting; first, because it shows what can be done in the way of surgery upon a woman of advanced years, but one also whose urine was markedly diabetic; and second, it proves how futile any operation would be short of high amputation in this class of cases. Both legs were removed below the knee joint, the vessels being very much degenerated, that of the left leg much more than the right, and as a result the sloughing was correspondingly much greater. No doubt, it would have been better to have amputated the left leg above the knee joint, as the vessel would have been larger and in better condition, but the added dangers of high amputations, together with the very satisfactory union which took place in the other leg, made me hope for an equally good result with the second amputation.

493 DELAWARE AVENUE.

